# Respiratory syncytial virus - associated intensive care unit admission in children in Southern China

**DOI:** 10.1186/1756-0500-6-447

**Published:** 2013-11-08

**Authors:** Qingli Zhang, Zhongqin Guo, Joanne M Langley, Zhenjiang Bai

**Affiliations:** 1 Department of Pediatrics, Xinhua Hospital, Shanghai Jiao Tong University School of Medicine, Shanghai, PR China; 2 Pediatric Intensive Care Unit, Children’s Hospital Affiliated to Soochow University, Suzhou, Jiangsu, PR China; 3 Department of Public Health, Ningxia Medical University, Yinchuan, PR China; 4 Department of Pediatrics, Canadian Center for Vaccinology, IWK Health Centre and Capital Health District, Dalhousie University, 5850 University Ave, Halifax, Nova Scotia B3K 6R8, Canada

**Keywords:** Respiratory syncytial virus, Intensive care, Child, Hospitalization

## Abstract

**Background:**

There are incomplete data on the global burden of viral lower respiratory tract infection, in particular the role of Respiratory Syncytial Virus, in children requiring health services.

**Findings:**

In this study set in a large urban area of southern China from 1 January 2007 to 31 December 2010, children 1 month to 14 years of age with RSV-associated “severe” or “very severe pneumonia” according to World Health Organization definitions, and meeting local criteria for admission to the pediatric intensive care unit, were followed for the course of their admission. The median age was 3 months and 79% (135/171) of children with RSV were under six months of age. All children needed supplemental oxygen, and 22% required mechanical ventilatory support. The mortality rate was 3.5%. In multivariate analysis, congenital heart disease and Trisomy 21 were associated with death.

**Conclusions:**

Children admitted to an intensive care unit with RSV-associated severe/very pneumonia in a large urban setting in southern China were most commonly ≤ six months old and almost one quarter of these had respiratory failure. The overall mortality rate was 3.5%. RSV vaccine strategies that would protect children from early infancy are urgently needed.

## Findings

## Background

RSV is the most common viral cause of acute lower respiratory tract infection (ALRI) in developed and developing countries [1,2]. RSV-associated ALRI manifests clinically as bronchiolitis and/or pneumonia. Children are admitted to hospital if respiratory distress prevents adequate feeding or if respiratory support is needed. In the developing world 3 to 9% of ALRI deaths are thought to be due to RSV [1].

In ambulatory and general ward settings in China RSV has been identified in 23 to 38% of children with ALRI [3-7], but there are little data on those ill enough to be admitted to an intensive care unit. In a study of severe and very severe community acquired pneumonia (S/VS-CAP) due to any cause in southern China we found that young age (<12 months) and congenital heart disease (CHD) were associated with pediatric intensive care unit (PICU) admission [8]. In this report we describe the subset of children with RSV, in order to determine the burden of morbidity and mortality with this common virus that could be alleviated by vaccines or other interventions.

## Study methods

The Children’s Hospital affiliated with Soochow University is the only referral centre for children less than 14 years of age in Suzhou city (pop 13 million, 2010), and has 25,000 pediatric admissions yearly. A prospective study of severe and very severe pneumonia, of any microbial etiology, was conducted in the PICU from 1 January 2007 to 31 December 2010; methods have been published [8]. In brief, eligible children were 1 month to 14 years of age, met WHO criteria for severe pneumonia or very severe pneumonia [9], and were enrolled within 24 hours of PICU admission at this hospital. Exclusionary criteria were refusal of parental consent to participate, or presence of pre-existing chronic illness, or a co-morbidity leading to admission.

Demographic and clinical information were collected by parental interview and review of the health record using standardized data collection forms. A chest radiograph and nasopharyngeal aspirate (NPA) for diagnosis of RSV, influenza virus A and B, para-influenza 1, 2, and 3 and adenovirus (Direct Immunofluorescence assay, Chemicon International – Millipore, Bellerica, Mass) were obtained. Subgroups of RSV (A, B) were not assessed. In this study we describe the subset of children with laboratory confirmed RSV infection.

Clinical management followed a predetermined protocol [8]. The criteria for assisted ventilation included inability to maintain a saturation of >90% on an FIO2 of >70%, apnea, hypercarbia with acidemia and clinical assessment of impending exhaustion [8].

Statistical analysis consisted of description of categorical variables, and comparisons of children with RSV that died or survived were made using multivariate analysis. P <0.05 was considered statistically significant. All analyses were performed using SPSS 13.0 software (IBM, Armonk, NY).

The study was approved by the Research Ethics Board of the Children’s Hospital affiliated with Soochow University and written informed consent was obtained from the parent/guardian of each child.

## Results

RSV was confirmed in 57% (171/295) of all children with viral severe or very severe community acquired pneumonia [8], and accounted for 24% of these PICU admissions (171/707) during the three year study period. Other viruses identified were influenza (19%; 56/295), para-influenza (11%; 32/295) and adenovirus (3.4%; 10/295). Viral co-infection occurred in 26 children; 21 of these included RSV infection.

The median age of RSV-infected PICU admissions was 3 months, with 79% (135/171) of patients being under 6 months of age (range 1 month-3 years). Males comprised 122 (71%) of cases. Pre-existing conditions were congenital heart disease (CHD) in 15% (n = 26), (4 of these patients also had Trisomy 21), followed by premature birth (n = 18; 10.5%). Nine children had Trisomy 21 alone as a risk factor, and a neurodevelopmental condition was present in 5.3% (n = 9). Over half of children were previously healthy with no risk factors for severe RSV illness (53%; 90/171).

Clinical features at presentation were similar over the three years, with all children presenting with cough, intercostal recession and tachypnea. Other symptoms (e.g. fever, lethargy) were variably present (data not shown). Although all children required supplemental oxygen, only 22% (37/171) required assisted ventilatory support. The average length of stay in the PICU was 5 days (range 3, 26 days).

The RSV mortality rate was 3.5% (Table 1). Death was associated with CHD (Odds Ratio (OR) 34.29, 95% confidence interval (CI) 3.82, 307.98, p = 0.00), and Trisomy 21 (OR 11.29, 95% CI 1.76, 72.39; p = 0.35). CHD was the only significant predictor of a PICU stay longer than 7 days) (OR 4.67, 95% CI 1.95,11.16; p < 0.001).

**Table 1:** Children 1 month through 14 years of age with severe/very severe community acquired pneumonia attributable to Respiratory Syncytial virus (RSV) requiring admission to a pediatric intensive care unit (PICU) (2006–2010) in a large urban center in China

***Year***	***ALRI admissions***	***Severe or very severe pneumonia (WHO criteria)***	***RSV severe or very severe pneumonia N (%)***	***Deaths associated with RSV pneumonia (%)***
2007-2008	399	123	34 (27.6)	1/34 (2.9%)
2008-2009	486	133	31 (23.3)	1/31 (3.2%)
2009-2010	539	192	46 (24)	2/46 (4.3%)
2010-2011	692	259	60 (23)	2/60 (3.3%)
*Total*	*2116*	*707*	*171 (24)*	*6/171 (3.5%)*

ALRI – acute lower respiratory tract infection; WHO = World Health Organization.

Mixed viral infections are not reported here.

**Figure 1:**
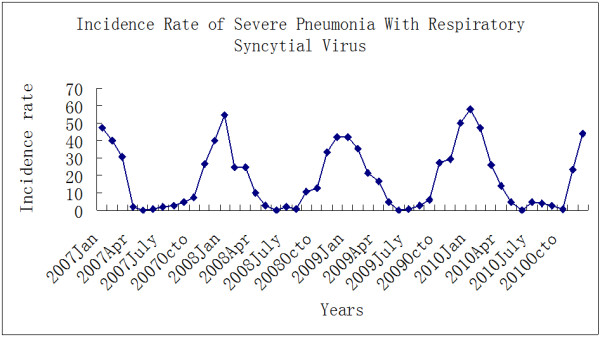
Seasonality of RSV-associated severe/very severe pneumonia admissions (n=171) to the pediatric intensive care unit (2007–2010).

The RSV season was consistent each year (Figure 1), with an annual epidemic from November to February.

## Discussion

In this study of pediatric intensive care unit admissions in a large Chinese city RSV was the etiologic agent in 24% of all episodes of severe/very severe pneumonia. This is likely an underestimate of the burden due to RSV because of several possible limitations. Firstly, given limited resources, we used an immunofluorescent viral diagnostic technique rather than a more sensitive, and expensive, molecular diagnostic technique (e.g. polymerase chain reaction, PCR). Secondly, PICU admission captures only the severe end of the spectrum of RSV illness. Finally, although our centre is the only care setting to which severely ill children would present in our area, it is possible that children could succumb before presenting to hospital, or not present for care because the family could not afford to pay 20% of the cost of hospital admission. The findings are also limited by our inability to perform subgroup typing (RSV A and B).

The 3.5% mortality rate we observed is comparable to that seen in developed countries rather than in the developing world [1,2], but higher than that found in a study in Hong Kong [10]. We found both congenital heart disease and Trisomy 21 were associated with mortality. In some western countries children at high risk for severe RSV illness, such as those with hemodynamically significant heart disease or premature birth may be offered passive immunization with the anti-RSV monoclonal antibody palivizumab [2,11]. While such a strategy, in which children receive monthly intramuscular injections of monoclonal antibody, is feasible in children with known risk factors in some countries, it is not feasible for most of the world’s children. Young age is a known vulnerability for severe RSV [2], and over this four-year study 79% of RSV cases were less than 6 months of age. Over half of the PICU admissions for RSV were in previously healthy children. These epidemiologic features point to the need for a strategy that would be available to all infants early in life. A vaccine given in late pregnancy or in early infancy would be necessary to benefit these young infants.

The November to February epidemics of RSV coincides with the colder months in this “humid, subtropical” region of China when the average temperature is 5 to 8 degrees Celsius. RSV activity increases in the winter months in temperate climates but may occur year around in equatorial areas and in tropical and subtropical areas or be more common in the wet season.

In summary we observed that RSV is a significant cause of life-threatening acute respiratory illness, particularly in the first year of life in this population. Passive immunization is too resource intensive to be feasible preventive strategy for RSV illness in this population. RSV vaccines are under investigation and are urgently needed to protect the very young infant.

## Competing interests

The authors declare that they have no competing interests.

## Authors’ contributions

QZ conceived the study. QZ and JML drafted the paper and all authors read and approved the final version. QZ and ZB collected data and ZG performed analyses.
